# Depression and prostate cancer risk: A Mendelian randomization study

**DOI:** 10.1002/cam4.3493

**Published:** 2020-10-07

**Authors:** Xiong Chen, Jianqiu Kong, Xiayao Diao, Jiahao Cai, Junjiong Zheng, Weibin Xie, Haide Qin, Jian Huang, Tianxin Lin

**Affiliations:** ^1^ Department of Urology Sun Yat‐sen Memorial Hospital Sun Yat‐sen University Guangzhou P. R. China; ^2^ Guangdong Provincial Key Laboratory of Malignant Tumor Epigenetics and Gene Regulation Sun Yat‐sen Memorial Hospital Sun Yat‐sen University Guangzhou P. R. China; ^3^ Department of Neurology Sun Yat‐sen Memorial Hospital Guangzhou P. R. China

**Keywords:** causality, depression, Mendelian randomization, prostate cancer

## Abstract

**Background:**

The association between depression and prostate carcinogenesis has been reported in observational studies but the causality from depression on prostate cancer (PCa) remained unknown. We aimed to assess the causal effect of depression on PCa using the two‐sample Mendelian randomization (MR) method.

**Methods:**

Two sets of genetics instruments were used for analysis, derived from publicly available genetic summary data. One was 44 single‐nucleotide polymorphisms (SNPs) robustly associated with major depressive disorder (MDD) and the other was two SNPs related with depressive status as ever depressed for a whole week. Inverse‐variance weighted method, weighted median method, MR‐Egger regression, MR Pleiotropy RESidual Sum, and Outlier test were used for MR analyses.

**Results:**

No evidence for an effect of MDD on PCa risk was found in inverse‐variance weighted (OR: 1.12, 95% CI: 0.97‐1.30, *p* = 0.135), MR‐Egger (OR 0.89, 95% CI: 0.29‐2.68, *p* = 0.833), and weighted median (OR: 1.08, 95% CI: 0.92‐1.27, *p* = 0.350). Also, no strong evidence for an effect of depressive status on PCa incidence was found using the inverse‐variance weighted method (OR 0.72, 95% CI: 0.35‐1.47, *p* = 0.364).

**Conclusions:**

The large MR analysis indicated that depression may not be causally associated with a risk of PCa.

## Graphical abstract

Using Mendelian randomization, we reveal the causality relationship from depression on prostate cancer. Both genetically predicted major depressive disorder and depressive status have no causality on prostate carcinogenesis.

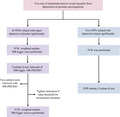

## 1 BACKGROUND

Prostate cancer (PCa) is the second most common cancer and the second most common cause of cancer‐related death in men; with an estimate of 191,930 new cases in 2020 in USA.^1^ Men have a high risk (11.2%) to be diagnosed with PCa in his lifetime.^2^ Advanced age, ethnicity, family history, smoking, are among the well‐established risk factors for PCa but till present, no modifiable risk factors have been established for PCa.^3^ To further reduce PCa burden, more attention should be paid to other potentially modifiable risk factors, such as psychiatric conditions.^4^

Depression is one of the leading causes of disability with more than 300 million incidences worldwide.^5^ It is an important risk factor for various diseases^6^, ^7^, ^8^ and has gradually become a research hotspot for prostate carcinogenesis and progression. Patients with depression seemed to be more vulnerable to PCa and have demonstrated poorer prognoses.^9^, ^10^, ^11^ A meta‐analysis suggested that depression was associated with a significantly increased risk of PCa incidence (1.37, 95% CI: 1.01‐1.86) and cancer‐specific mortality (adjusted RR: 1.87, 95% CI: 1.62‐2.16).^12^ Positive association between a history of depression and PCa was reported in a study which followed 3177 cancer‐free adults for 24 years, but confidence bounds included the null.^13^ Due to potential biases such as confounders or reverse causation, the association between depression and PCa has not been systematically examined; thus, whether depression plays a causal role in the development of PCa remains undiscerned.

The use of Mendelian randomization (MR) can overcome these biases by using genetic variants indexing exposure to inter the causality of the risk factors related to the disease.^14^, ^15^ If an exposure such as major depressive disorder (MDD) causally influences an outcome such as PCa, then, a variant that affects MDD should be expected to influence PCa to a proportional degree. But horizontal pleiotropy which means separate pathway by which this variant can affect PCa should be excluded first.

In this study, we applied a two‐sample MR using summary statistics from large scale genome‐wide association studies (GWAS) of MDD, depressive status, and PCa, to reveal the causal effect of depression on the risk of prostate carcinogenesis.

## 2 METHODS

Due to such a re‐analysis of previously collected and published data, no additional ethics approval was needed.

### 2.1 Genetic variants associated with MDD and depressive status

**FIGURE 1:**
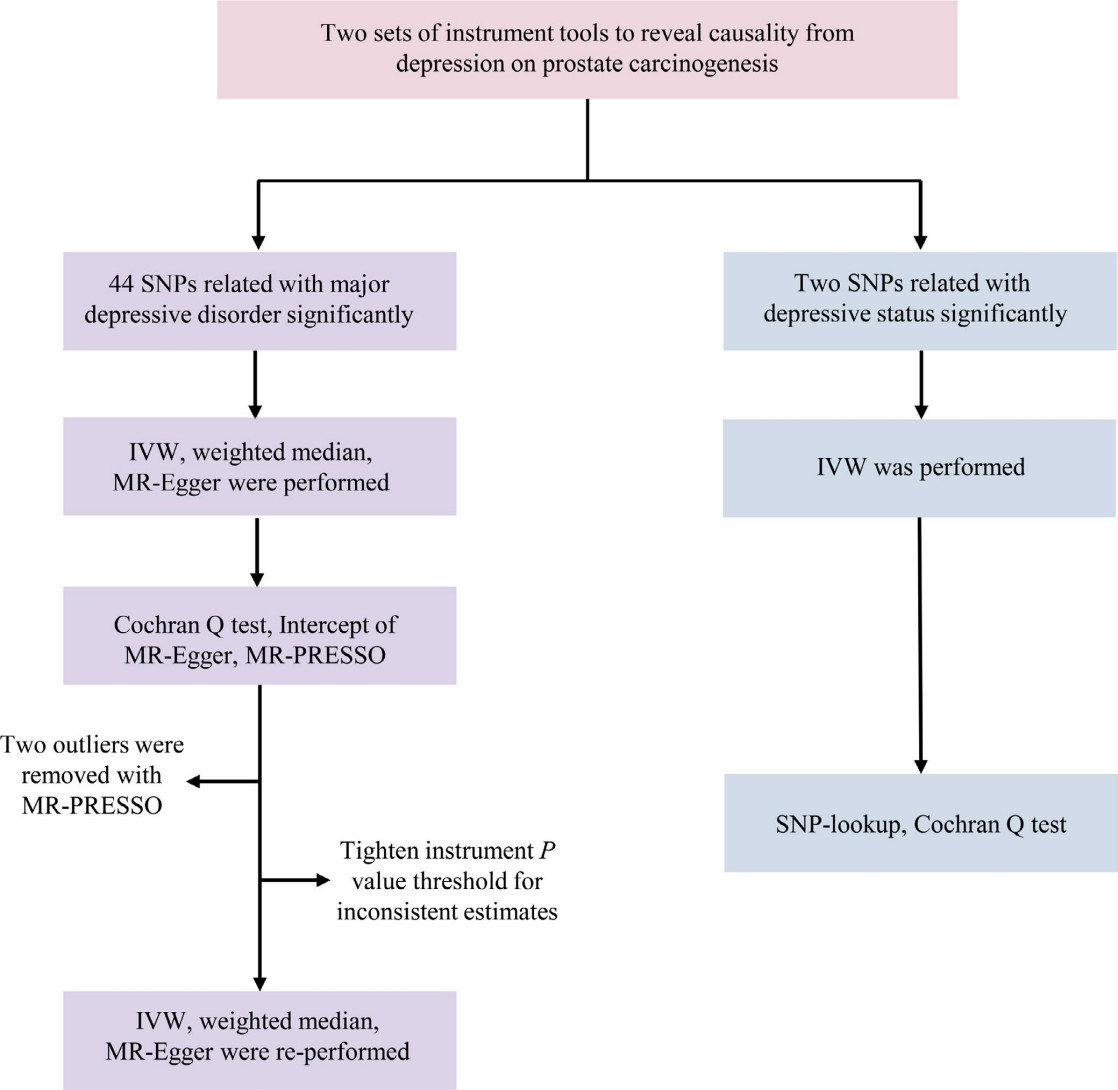
Workflow of Mendelian randomization study revealing causality from depression on prostate carcinogenesis. IVW, inverse variance weighted; MR, Mendelian randomization; MR‐PRESSO, MR Pleiotropy RESidual Sum and Outlier; SNP, single‐nucleotide polymorphisms.

An overview of the study design is presented in Figure 1. There were two sets of genetic instruments used to reveal the causality from depression on PCa. The primary genetic instruments were derived from the summary data of a recent GWAS meta‐analysis on MDD which contained seven MDD cohorts.^16^ Totally, 135,458 MDD cases and 344,901 controls were analyzed in the meta‐analysis. 44 single‐nucleotide polymorphisms (SNPs) were reported significantly related with MDD (*p* < 5 × 10^−8^, linkage disequilibrium [LD] *r* ^2^ < 0.01). Details of the 44 SNPs are listed in the Table S1. These SNPs explained 0.23% of the variability in MDD.^7^ The *F*‐statistics was 156, larger than the conventional value of 10, indicating that the instruments had strong potential to predict MDD.^17^

To avoid violation from the variation of depression severity, we constructed another set of genetic instruments based on the depressive status of a recent GWAS from the Neale Lab consortium which included 125,193 cases who experienced ever‐depressed conditions for a whole week and 113,333 control cases. To include more SNPs that contributed to depressive status, a more relaxed threshold (*p* < 5 × 10^−7^) was used; and which had been previously used in many psychiatric MR researches.^18^

The LD (*r* ^2^ < 0.01) were also evaluated. As a result, two SNPs (rs11948151 and rs3025649) were identified. These two SNPs explained 0.86% of the variability in depressive status, and the *F*‐statistics was 237, supporting that the instruments confidently predicted depressive status.^17^

### 2.2 GWAS summary data for PCa

The GWAS summary data for PCa were obtained from the PRACTICAL consortium.^19^ Totally, there were 79,148 PCa cases and 61,106 control cases. We retrieved summary data from the PRACTICAL, and each of the 44 SNPs associated with MDD as well as the two SNPs associated with depressive status (including the effects of each of the SNPs on PCa; effect sizes and standard errors) were extracted. SNP rs62099069 from the 44 SNPs was removed for being palindromic with intermediate allele frequency.

### 2.3 GWAS summary data for PCa risk factors

To reveal the mediating effect of depression on PCa through other risk factors, inverse variance weighted (IVW) was performed to estimate the association between MDD/depressive status and other known PCa risk factors. Well‐accepted risk factors included BMI, smoking, and alcohol consumption. Genome‐wide association studies summary data for these phenotypes were extracted from the Tobacco and Genetics consortium,^20^ Genetic Investigation of ANthropometric Traits consortium^21^ and Neale Lab consortium. Details of all GWASs included in our study are represented in Table 1.

**TABLE 1:** Details of the GWASs included in the Mendelian randomization

Consortium	Phenotype	Participants	Web source
A meta‐analysis of GWAS	Major depressive disorder	480,359	https://doi.org/10.1038/s41588‐018‐0090‐3
Neale Lab Consortium	Ever depressed for a whole week	238,526	http://www.nealelab.is/uk‐biobank
PRACTICAL	Prostate cancer	140,254	http://practical.icr.ac.uk/blog/
TAG	Smoking	74,053	www.med.unc.edu/pgc/results‐and‐downloads
GIANT	Body mass index	339,224	https://portals.broadinstitute.org/collaboration/giant
Neale Lab Consortium	Alcohol consumption	336,965	http://www.nealelab.is/uk‐biobank

Abbreviations: GIANT, Genetic Investigation of ANthropometric Traits consortium; TAG, Tobacco and Genetics consortium.

### 2.4 Statistical analyses

After harmonization of the effect alleles across the GWASs of MDD and PCa, we used several MR approaches to determine MR estimates of MDD for PCa, namely the IVW, weighted median, and MR‐Egger. Multiple approaches were used as they have different underlying assumptions for horizontal pleiotropy. Inverse variance weighted meta‐analysis of the Wald ratio for individual SNPs, which assumes that instruments can affect the outcome only through the exposure of interest and not by any alternative pathway, was used as the main outcome.^22^ MR‐Egger and weighted median methods were used to complement IVW estimates as these approaches could provide more robust estimates in a broader set of scenarios but are less efficient (wider CIs). If estimates of these approaches in our study were inconsistent, a tighten instrument *p* value threshold was set.^23^

Sensitivity analysis has been pivotal in MR studies to detect underlying pleiotropy and the heterogeneity for MR estimates can be severely violated. We used heterogeneity markers (Cochran *Q*‐derived *p* < 0.05) from the IVW approach to represent potential horizontal pleiotropy. The intercept obtained from the MR‐Egger regression was an indicator for directional pleiotropy (*p* < 0.05 was considered as the presence of directional pleiotropy).^24^ MR‐Pleiotropy Residual Sum and Outlier methods (MR‐PRESSO) were also used to assess and correct horizontal pleiotropy.^23^ MR‐PRESSO includes three components: (a) detection of horizontal pleiotropy; (b) correction for horizontal pleiotropy via outlier removal; (c) testing of significant differences in the causal estimates before and after correction for outliers. It is less biased and has better precision than IVW, MR‐Egger when the percentage of horizontal pleiotropy variants is smaller than 10%.^25^ Leave‐one‐out analysis was also performed to evaluate whether the MR estimate was driven or biased by a single SNP.

Since only two SNPs were found to be significantly and independently associated with depressive status, only the IVW method was applied. Despite this, we also conducted pleiotropy assessment on potential confounders using a website tool Single Nucleotide Polymorphisms Annotator (https://snipa.helmholtz‐muenchen.de/snipa3/).^26^ Analyses were implemented by the package TwoSampleMR (version 0.4.25) and MRPRESSO (version 1.0) in R (version 3.6.1).

## 3 RESULTS

### 3.1 Causal effect from MDD to PCa

**FIGURE 2:**
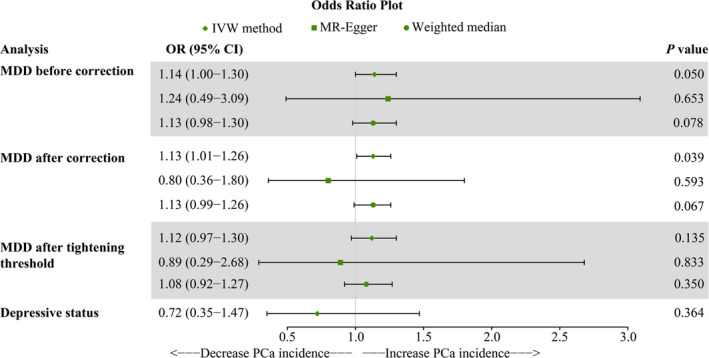
Odds ratio plot for MDD and depressive status. MDD, major depressive disorder; OR, odds ratio

Using the 43 MDD‐related SNPs, we found weak evidence of a potential causal effect of MDD on the risk of PCa at borderline statistical significance (OR = 1.14, 95% CI = 1.00‐1.30, *p* = 0.050). Meanwhile, similar risk estimates were gained using the MR‐Egger regression (OR = 1.24, 95% CI = 0.49‐3.09, *p* = 0.653) and weighted median approaches (OR = 1.13, 95% CI = 0.98‐1.30, *p* = 0.078); though the association was not statistically significant. However, heterogeneity was observed with a Cochran *Q*‐test derived *p* value as 2.90 × 10^−7^ of MR‐Egger and *p* value as 4.58 × 10^−7^ of IVW. MR‐PRESSO also presented a similar result (*p* value in the global heterogeneity test <0.001). With two outliers removed (rs115507122 and rs11643192), the MR approaches were re‐applied to evaluate the relationship between MDD and PCa (Figure 2). With the IVW method, MDD increased risk for PCa significantly (OR 1.13, 95% CI: 1.01‐1.26, *p* = 0.039), while opposing results were observed using the MR‐Egger approach (OR: 0.80, 95% CI: 0.36‐1.80, *p* = 0.593). Since the MR estimates of MR‐Egger and IVW were inconsistent, we tighten the instrument *p* value threshold to 1 × 10^−8^ and 27 SNPs were used as instrument tools.^23^ The MR estimates turned nonsignificant, indicating that a genetically predicted increase in MDD was not significantly associated with PCa risk (Figure 2). The MR regression slopes and individual causal estimates of each of the 27 SNPs are illustrated in Figures S1 and S2. Furthermore, there was no evidence for a significant intercept (intercept = 0.008; SE = 0.018. *p* = 0.681), indicating that there was no directional pleiotropy observed.

Indeed, no obvious causal effect was observed between MDD and PCa. No single SNP was strongly violating the overall effect of MDD on PCa in the leave‐one‐out sensitivity analysis (Figure S3). Further, the funnel plot was symmetry, indicating no pleiotropy (Figure S4).

### 3.2 Causal effect from depressive status to PCa

Two SNPs that were significantly and independently associated with depressive status were identified (rs11948151 and rs3025649). The result of IVW indicated that depressive status had no causality on PCa (OR = 0.72, 95% CI = 0.35‐1.47, *p* = 0.364). The Cochran *Q*‐test derived *p* value was 0.363, indicating that no obvious heterogeneity was observed.

### 3.3 Causal effect from MDD and depressive status on potential PCa risk factors

To identify whether the MR association between genetically determined depression and PCa was violated through pleiotropic pathways relating to PCa, we investigated the relationship between depression and several PCa risk factors such as smoking, alcohol consumption, body mass index, using the IVW method. No causal effects were observed from MDD and depressive status on potential PCa risk factors (Tables 2 and 3).

**TABLE 2:** Mendelian randomization estimates of the associations from major depressive disorder on common risk factors

Outcome	Causal effect (95% CI)	*p* value
Ever vs never smoker	1.14 (0.95‐1.37)	0.169
Former vs current smoker	1.00 (0.79‐1.27)	0.976
Cigarettes smoked per day	0.51 (0.18‐1.42)	0.197
Body mass index	1.05 (0.91‐1.21)	0.336
Alcohol drinker status: never vs ever	1.00 (0.99‐1.00)	0.446

**TABLE 3:** Mendelian randomization estimates of the associations from depressive status on common risk factors

Outcome	Causal effect (95% CI)	*p* value
Ever vs never smoker	1.16 (0.14‐9.72)	0.892
Former vs current smoker	1.09 (0.12‐10.40)	0.938
Cigarettes smoked per day	0.90 (0.0010‐928.30)	0.976
Body mass index	0.95 (0.48‐1.89)	0.885
Alcohol drinker status: never vs ever	1.01 (0.97‐1.04)	0.763

## 4 DISCUSSION

We applied a two‐sample MR approach to comprehensively evaluate whether depression causally influences PCa incidence and found no clear evidence to support the causal role of genetically predicted depression on the risk of PCa.

Up to now, relationship between depression and PCa has remained unclarified.^12^, ^13^, ^27^ Most previous epidemiology studies were case‐control designed and failed to clarify the causality with blurred temporal order. Even in prospectively observational studies, the reverse effect still existed for some diagnosed cancer and undiagnosed subclinical cancer could give rise to depression; thus, depression may not act as predictors of cancer development and survival but may instead be an outcome from cancer.^28^ Besides, all previous observational studies were hard to avoid violations from confounding risk factors, while in this present study, by applying MR methods, we could confidently reveal causality apart from bias due to better study design.

A recent meta‐analysis indicated that diagnosed depression and depressive status might play a different role in PCa incidence.^12^ To address this, we performed MR estimates using two sets of genetic instruments and results indicating that both MDD and depressive status had no causality on PCa incidence.

Since there were only two SNPs that were significantly and independently associated with depressive status, sensitivity studies such as MR‐Egger and MR‐PREESO were not suitable to conduct. Despite this, we performed functional annotations for the two depressive status‐related SNPs using a website tool Single Nucleotide Polymorphisms Annotator.^26^ SNP rs3025649 was found to associate with diabetes mellitus. The relationship between diabetes mellitus and PCa has also attracted great interest. Chen, M. reported that Diabetes was associated with a higher risk of PCa detection (OR = 1.51, 95% CI: 1.12‐2.05, *p* = 0.007) retrospectively analyzing 2032 Chinese patients.^29^ However, Dankner, R. and Miller, E. A., respectively, reported that diabetes influenced the PCa screening strategies rather than reduce the incidence.^30^, ^31^ From observational studies, evidence prefer to support that diabetes does not increase the risk of PCa; thus, our finding might not be violated by diabetes. SNP rs11948151 was found to be associated with interleukin‐7α (IL‐7α). Elevated IL‐7 expression has been reported in PCa tissues and was found to be closely related with poor prognosis.^32^, ^33^ Min A. Seol and his colleagues found that IL‐7 contributes to the invasiveness of PCa on cellular level experiments through the epithelial‐mesenchymal transition.^34^ Qu H reported that the IL‐7/IL‐7 receptor axis probably activates the AKT/NF‐κB pathway and upregulates the expression of MMP‐3/7 to increase PCa invasion and migration.^35^ However, no evidence yet supported that IL‐7 could increase PCa incidence. When removing rs11948151, the MR estimate remained null, indicating that our findings were not violated by pleiotropy.

Though our findings suggested that no causality between depression with PCa incidence, it was possible that depression might have effect on the progression of PCa,^36^ which was not illustrated in the scope of the current study. There are several underlying pathways between depression and PCa that have been widely accepted, both at biological and behavioral levels.^12^ Depression can directly influence endocrine and immune processes.^37^ Dysregulation of the hypothalamic‐pituitary‐adrenal axis has been implicated as a potential mediator of PCa. Depression has found to suppress the activity of natural killer cells and DNA repair enzymes, which play pivotal roles in cancer defense process.^28^ Depression was also associated with the inflammatory markers (i.e., interleukin 1, 6; C‐reactive protein, while in our study, the role of IL‐7 might be more significant).^38^ There were also several indirectly pathways leading to cancer through lifestyle‐related factors such as smoking, alcohol abuse, and obesity.^39^

Our study has several strengths. First, using the MR design, our study can simulate randomized controlled trials in observational settings. Randomized controlled traits are widely accepted in studying causality but costs are rather expensive and frequently impractical to conduct. But MR studies can effectively avoid confounding bias for SNPs were randomly assigned at conception. Compared to other observational studies, MR can also avoid the reverse causal effect. Second, our findings may influence health care policies for depression and PCa. Given the high prevalence of depression and PCa in the general population, revealing causality between depression and PCa influences public health policies about early prevention and timely intervention.^2^ Our finding implied that strengthening screening PCa in patients with genetically predicted depression may be useless. More attention should be paid to reveal association between environment determined depression and prostate carcinogenesis or depression and prognosis of PCa.

However, several limitations were also present. First, all GWASs data came from European population. Whether our described findings would be consistent in other populations remained to be investigated. Second, we should pay attention to the variety among patients with PCa. Depression might have causality on a certain kind of PCa. A broader study containing subgroups of PCa can be considered in the future.

## 5 CONCLUSION

This is the first MR study to explore the causality from depression on PCa. Our MR analysis does not support the hypothesis that depression could increase PCa incidence.

## CONFLICT OF INTEREST

The authors declare no conflict of interest.

## AUTHOR CONTRIBUTIONS

Conceptualization, Xiong Chen, Jianqiu Kong and Tianxin Lin; Formal analysis, Xiong Chen; Methodology, Xiong Chen and Jiahao Cai; Resources, Xiong Chen and Jianqiu Kong; Supervision, Tianxin Lin; Visualization, Xiong Chen and Jianqiu Kong; Writing ‐ original draft, Xiong Chen; Writing ‐ review & editing, Jianqiu Kong, Xiayao Diao, Jiahao Cai, Junjiong Zheng, Weibin Xie, Haide Qin, Jian Huang and Tianxin Lin.

## Supporting information

Supplementary Material

## Data Availability

All data used in the current study are publicly available GWAS summary data.
