# Supplementary material for: Depression and prostate cancer risk: A Mendelian randomization study
Source: Cancer Med. 2020 Oct 7;9(23):9160–7. doi: 10.1002/cam4.3493 (PMC7724297; doi:10.1002/cam4.3493)
Supplement: Supplementary file 1 — Supplementary Material [file CAM4-9-9160-s001.docx]

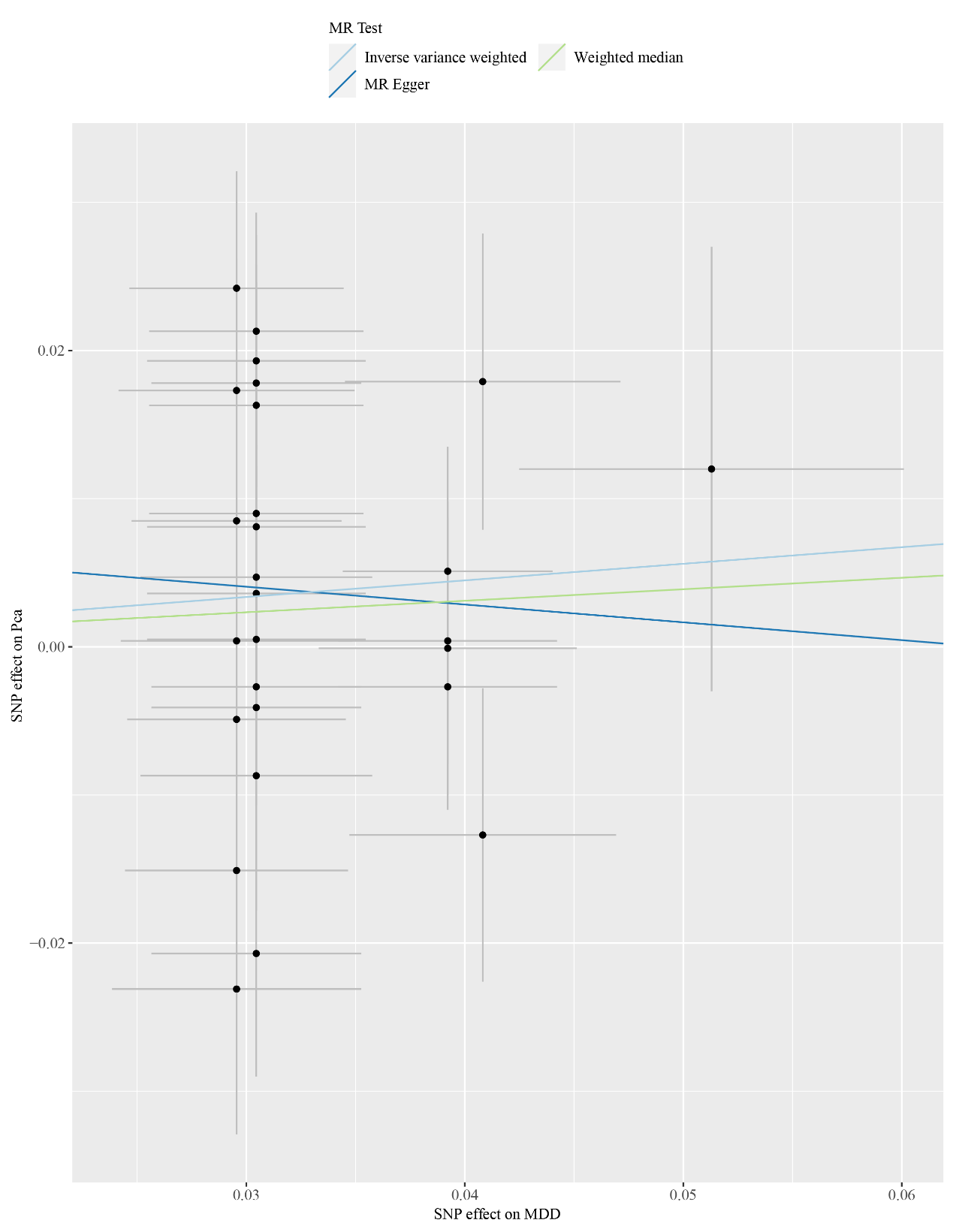


Figure S1. Scatter plot of SNPs associated with MDD and their risk of prostate cancer after outliers removal with MR-PRESSO and tightening instrument *P* value threshold.


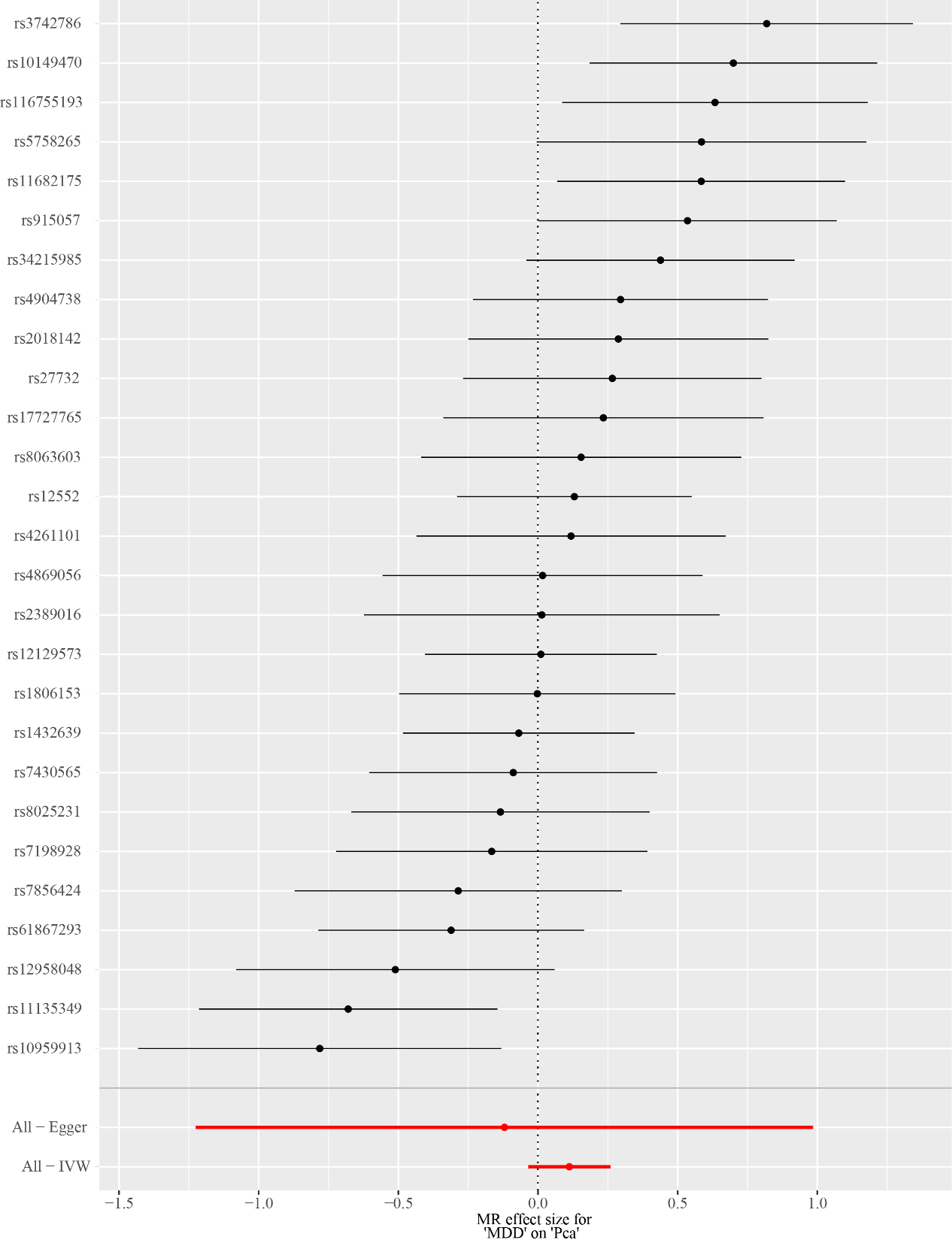


Figure S2. Forest plot of SNPs associated with MDD and their risk of prostate cancer after outliers removal with MR-PRESSO and tightening instrument *P* value threshold.


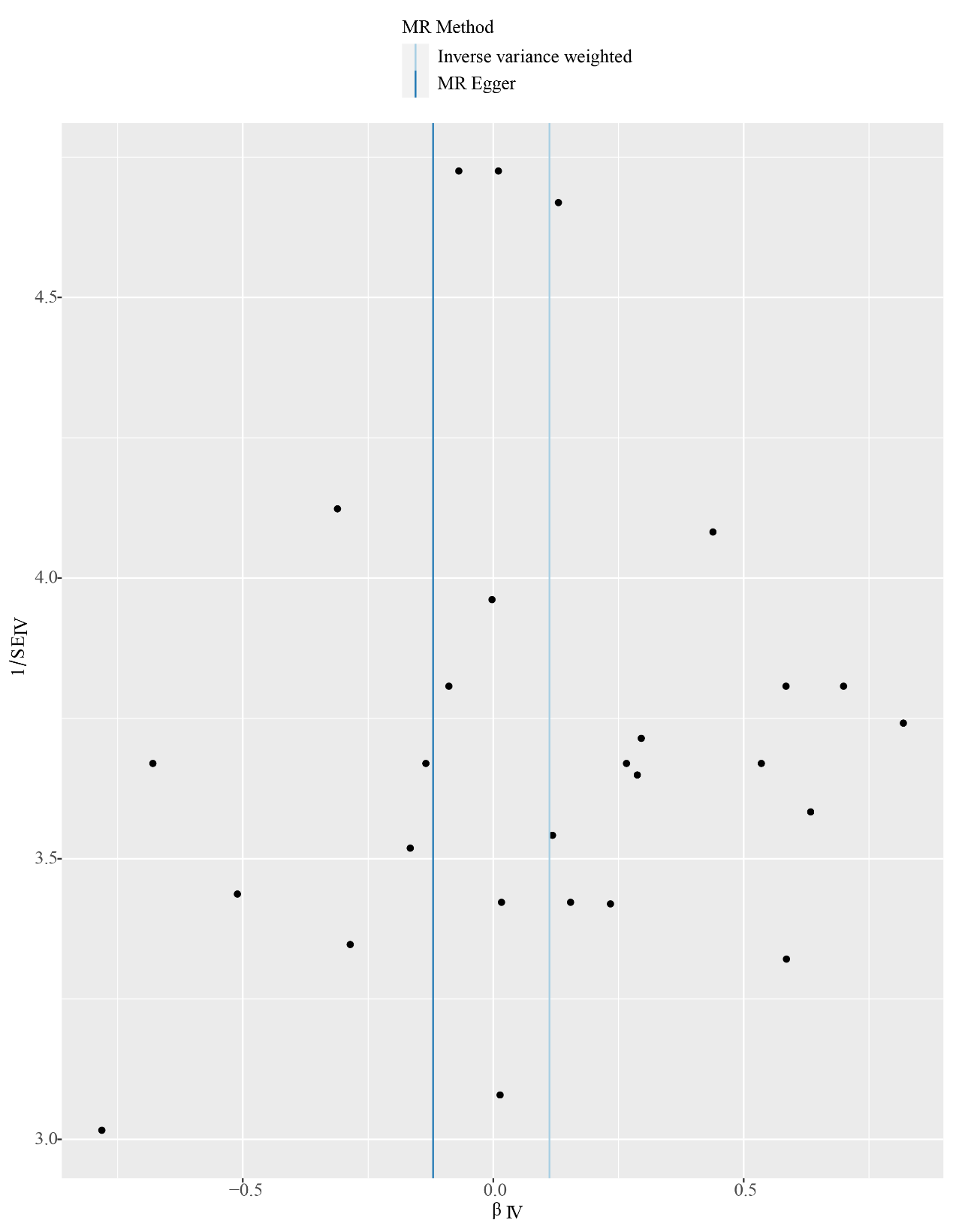
Figure S3. Funnel plot of SNPs associated with MDD and their risk of prostate cancer after outliers removal with MR-PRESSO and tightening instrument *P* value threshold.


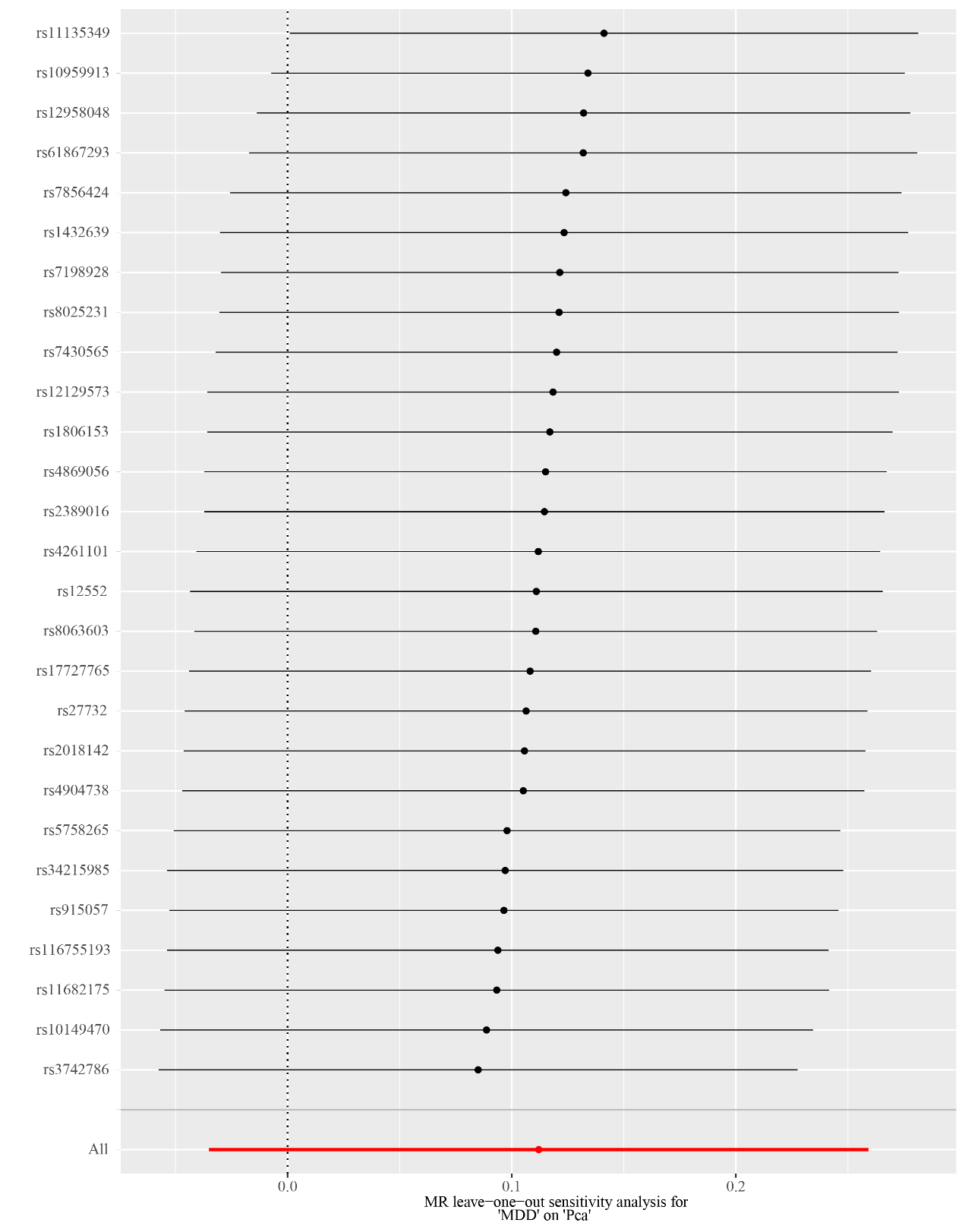


Figure S4. Leave-one-out of SNPs associated with MDD and their risk of prostate cancer after outliers removal with MR-PRESSO and tightening instrument *P* value threshold.

Supplementary Table 1. 44 SNPs associated with major depressive disorder

| SNP | Chromosome | Position | Effect  allele | Other allele | Frequency | Beta | Se | P | Sample  size |
| --- | --- | --- | --- | --- | --- | --- | --- | --- | --- |
| rs12129573 | 1 | 73768366 | A | C | 0.3671 | 4.00E-04 | 0.0083 | 0.9568 | 140,254 |
| rs1432639 | 1 | 72813218 | A | C | 0.6187 | -0.0027 | 0.0083 | 0.746 | 140,254 |
| rs159963 | 1 | 8504421 | A | C | 0.5671 | 0.0051 | 0.0085 | 0.5495 | 140,254 |
| rs2389016 | 1 | 80799329 | T | C | 0.2744 | 4.00E-04 | 0.0096 | 0.9632 | 140,254 |
| rs4261101 | 1 | 90796053 | A | G | 0.3543 | -0.0036 | 0.0086 | 0.6699 | 140,254 |
| rs9427672 | 1 | 1.98E+08 | A | G | 0.2187 | -0.018 | 0.0101 | 0.07444 | 140,254 |
| rs11682175 | 2 | 57987593 | T | C | 0.5333 | -0.0178 | 0.008 | 0.02626 | 140,254 |
| rs1226412 | 2 | 1.57E+08 | T | C | 0.7859 | -0.005 | 0.01 | 0.6207 | 140,254 |
| rs9862324 | 3 | 44433910 | T | C | 0.669 | -0.0085 | 0.0086 | 0.326 | 140,254 |
| rs7430565 | 3 | 1.58E+08 | A | G | 0.5674 | 0.0027 | 0.008 | 0.7403 | 140,254 |
| rs34215985 | 4 | 42047778 | C | G | 0.2135 | -0.0179 | 0.01 | 0.07425 | 140,254 |
| rs27732 | 5 | 87992576 | A | G | 0.4167 | -0.0081 | 0.0083 | 0.3302 | 140,254 |
| rs2018142 | 5 | 1.04E+08 | A | C | 0.5229 | -0.0085 | 0.0081 | 0.2922 | 140,254 |
| rs116755193 | 5 | 1.24E+08 | T | C | 0.3786 | -0.0193 | 0.0085 | 0.02314 | 140,254 |
| rs11135349 | 5 | 1.65E+08 | A | C | 0.4504 | 0.0207 | 0.0083 | 0.01254 | 140,254 |
| rs4869056 | 5 | 1.67E+08 | A | G | 0.6311 | -5.00E-04 | 0.0089 | 0.9554 | 140,254 |
| rs115507122 | 6 | 30737591 | C | G | 0.172 | -0.0526 | 0.0106 | 7.09E-07 | 140,254 |
| rs9402472 | 6 | 99566521 | A | G | 0.2222 | -0.006 | 0.0099 | 0.5395 | 140,254 |
| rs10950398 | 7 | 12264871 | A | G | 0.4074 | 0.011 | 0.0081 | 0.1762 | 140,254 |
| rs12666117 | 7 | 1.09E+08 | A | G | 0.4652 | -0.0034 | 0.008 | 0.6718 | 140,254 |
| rs1354115 | 9 | 2983774 | A | C | 0.6308 | 0.0092 | 0.0089 | 0.3049 | 140,254 |
| rs10959913 | 9 | 11544964 | T | G | 0.765 | -0.0231 | 0.0098 | 0.01801 | 140,254 |
| rs7856424 | 9 | 1.2E+08 | T | C | 0.2896 | 0.0087 | 0.0091 | 0.3379 | 140,254 |
| rs7029033 | 9 | 1.27E+08 | T | C | 0.0796 | -0.0189 | 0.0151 | 0.2121 | 140,254 |
| rs61867293 | 10 | 1.07E+08 | T | C | 0.206 | 0.0127 | 0.0099 | 0.2009 | 140,254 |
| rs1806153 | 11 | 31850105 | T | G | 0.225 | -1.00E-04 | 0.0099 | 0.9951 | 140,254 |
| rs4074723 | 12 | 23947737 | A | C | 0.401 | -0.0168 | 0.0091 | 0.06559 | 140,254 |
| rs4143229 | 13 | 44327799 | A | C | 0.9189 | -0.0093 | 0.0148 | 0.5284 | 140,254 |
| rs12552 | 13 | 53625781 | A | G | 0.4329 | 0.0051 | 0.0084 | 0.5459 | 140,254 |
| rs4904738 | 14 | 42179732 | T | C | 0.555 | -0.009 | 0.0082 | 0.2716 | 140,254 |
| rs915057 | 14 | 64686207 | A | G | 0.4302 | -0.0163 | 0.0083 | 0.05126 | 140,254 |
| rs3742786 | 14 | 75373011 | A | G | 0.4488 | 0.0242 | 0.0079 | 0.002278 | 140,254 |
| rs10149470 | 14 | 1.04E+08 | A | G | 0.4949 | -0.0213 | 0.008 | 0.007901 | 140,254 |
| rs8025231 | 15 | 37648402 | A | C | 0.5519 | 0.0041 | 0.0083 | 0.6174 | 140,254 |
| rs8063603 | 16 | 6310645 | A | G | 0.6568 | -0.0047 | 0.0089 | 0.6002 | 140,254 |
| rs7198928 | 16 | 7666402 | T | C | 0.6256 | -0.0049 | 0.0084 | 0.5638 | 140,254 |
| rs7200826 | 16 | 13066833 | T | C | 0.2605 | -0.0089 | 0.0096 | 0.3528 | 140,254 |
| rs11643192 | 16 | 72214276 | A | C | 0.4027 | -0.0234 | 0.0082 | 0.004284 | 140,254 |
| rs17727765 | 17 | 27576962 | T | C | 0.9147 | -0.012 | 0.015 | 0.4247 | 140,254 |
| rs62099069 | 18 | 36883737 | A | T | 0.4246 | -0.0097 | 0.0084 | 0.2505 | 140,254 |
| rs11663393 | 18 | 50614732 | A | G | 0.4713 | 0.0079 | 0.008 | 0.3242 | 140,254 |
| rs1833288 | 18 | 52517906 | A | G | 0.7116 | 0.0192 | 0.0095 | 0.04256 | 140,254 |
| rs12958048 | 18 | 53101598 | A | G | 0.3223 | -0.0151 | 0.0086 | 0.07798 | 140,254 |
| rs5758265 | 22 | 41617897 | A | G | 0.2832 | 0.0173 | 0.0089 | 0.05145 | 140,254 |
